# Copper Dyshomeostasis Affects α-Synuclein Clearance Mechanisms in Parkinson’s Disease: Insights from In Vitro Models and Translational Evidence

**DOI:** 10.3390/ijms27072993

**Published:** 2026-03-25

**Authors:** Debora Musarò, Marco Greco, Martina Lanza, Marina Damato, Michele Maffia

**Affiliations:** Department of Experimental Medicine, University of Salento, Via Lecce—Monteroni, 73100 Lecce, Italy; debora.musaro@unisalento.it (D.M.); marco.greco@unisalento.it (M.G.); martina.lanza@unisalento.it (M.L.); marina.damato@unisalento.it (M.D.)

**Keywords:** copper, Parkinson’s disease, alpha-synuclein, autophagy, ubiquitin proteasome system

## Abstract

Parkinson’s disease (PD) is characterized by the progressive degeneration of dopaminergic neurons and the accumulation of α-synuclein-rich inclusions, largely resulting from impaired protein clearance mechanisms. Copper is an essential redox-active metal in the central nervous system (CNS), but alterations in its homeostasis can promote oxidative stress, mitochondrial dysfunction, and proteostatic failure. In vitro studies indicate that copper can promote α-synuclein misfolding, enhance oxidative stress, and interfere with both the ubiquitin–proteasome system (UPS) and the autophagy–lysosome pathway (ALP). In this review, we critically evaluate mechanistic evidence from cellular models, integrating available animal and clinical data to assess the biological significance of copper-mediated impairment of α-synuclein clearance. We highlight the current research, identify methodological limitations, and discuss whether copper imbalance acts as a primary pathogenic trigger or as a disease-modifying amplifier of proteostatic failure. Furthermore, we consider the translational implications of selectively modulating intracellular copper pools as a therapeutic strategy in PD. Finally, we will highlight unresolved issues, methodological limitations, and emerging targeted therapeutic prospects.

## 1. Introduction

Parkinson’s disease (PD) is a progressive neurodegenerative disorder characterized by the selective loss of dopaminergic neurons in the substantia nigra pars compacta (SNpc) and the accumulation of intracellular protein inclusions known as Lewy bodies (LBs), primarily composed of α-synuclein [1]. α-synuclein is a presynaptic protein whose misfolding, aggregation, and impaired clearance are widely recognized as key pathogenic events in both familial and sporadic forms of PD [2]. While genetic mutations and environmental factors contribute to α-synuclein pathology, increasing evidence suggests that disturbances in metal homeostasis play a critical modulatory role in disease progression [3].

Among transition metals, copper occupies a unique position in the central nervous system (CNS) [4]. As an essential trace element, copper is required for numerous enzymatic processes, including mitochondrial respiration, antioxidant defense, and neurotransmitter synthesis [5]. However, copper is also a redox-active metal, and its intracellular levels must be tightly regulated to prevent oxidative damage [6]. Alterations in copper homeostasis have been reported in PD patients, with abnormal copper distribution observed in brain tissue, cerebrospinal fluid, and peripheral biological fluids [5]. These findings support the notion that copper dyshomeostasis may contribute to neuronal vulnerability and protein misfolding in PD.

α-Synuclein is particularly susceptible to metal-induced conformational changes [7]. Copper ions can directly bind α-synuclein at specific amino acid residues, promoting structural rearrangements that favor oligomerization and aggregation [8]. In addition, copper-mediated redox cycling enhances the generation of reactive oxygen species (ROS), leading to oxidative modifications of α-synuclein that further increase its aggregation propensity and toxicity [9]. While these interactions have been extensively studied from a biophysical and biochemical perspective, their impact on the cellular mechanisms responsible for the protein clearance remains incompletely understood.

Protein homeostasis in neurons relies primarily on the ubiquitin–proteasome system and the autophagy–lysosome pathway (ALP), including chaperone-mediated autophagy (CMA), all of which are critically involved in the degradation of α-synuclein [10]. Dysregulation of these pathways is a hallmark of PD and is closely associated with α-synuclein accumulation [11]. Evidence from in vitro models indicates that copper overload can interfere with multiple components of the cellular clearance machinery, impairing proteasomal activity, altering lysosomal function, and disrupting autophagic flux [12,13].

The diversity of experimental approaches and model systems has generated fragmented and sometimes conflicting data, underscoring the need for a comprehensive and critical synthesis of current knowledge.

In this review, we critically evaluate how copper dyshomeostasis influences α-synuclein clearance mechanisms in PD, with particular emphasis on mechanistic insights from in vitro models integrated with available in vivo and clinical evidence. Human and animal data are discussed as contextual frameworks to interpret in vitro findings. We focus on the molecular interactions between copper and α-synuclein, the effects of copper on proteasomal and autophagic pathways, and the relevance of these findings for understanding PD pathogenesis and identifying novel therapeutic strategies targeting metal homeostasis and proteostasis.

## 2. Copper Homeostasis in the Brain

Copper is a trace element and the third-most abundant transition metal of the body [4]. It is required in several biological processes, including energy metabolism, antioxidant defense, iron metabolism, and neurotransmitter synthesis [5]. The high redox potential of the Cu(II)/Cu(I) system is exploited for oxidation reactions, such as the generation of superoxides by copper–zinc superoxide dismutase (SOD1) and the production of catechols by tyrosinase [14]. In the nervous system, copper is involved in myelination, neuronal proteostasis [15] and modulation of synaptic activity through the interaction with various neurotransmitter receptors via metalloallostery [6,16]. However, because of its ability to generate toxic ROS, intracellular copper levels must be tightly regulated within the cell [17].

Copper is acquired through diet and transported into cells primarily via copper transporter 1 (CTR1), while divalent copper is absorbed by the divalent metal transporter 1 (DMT1) [18]. Here, the metal is sequestered by glutathione (GSH) or stored by metallothioneins (MTs), cysteine-rich proteins with high affinity for the metal. Copper is then distributed to its specific targets by copper-chaperones [19]. Atox1 transports copper to the P-type ATPases ATP7A and ATP7B, which regulate copper incorporation into cuproenzymes and cellular efflux [20,21].

Copper is involved in several biological functions, including respiration, ROS detoxification, neurotransmitter synthesis and degradation. In the nervous system, it can also be released into synaptic clefts to modulate neuronal excitability. Together with the liver, the brain accumulates the highest amounts of the metal; within the organ, the highest copper concentration was found in the SNpc [22]. Copper participates in neuronal signaling and can be released into the synaptic cleft in a Ca^2+^-dependent manner [23]. It also modulates the activity of NMDA and GABA receptors, as well as voltage-gated ion channels, thereby influencing neuronal excitability [24,25].

Copper interacts with synaptic proteins such as amyloid precursor protein (APP) and prion protein (PrP), suggesting a larger role in modulating protein conformation and aggregation processes relevant to neurodegeneration [26].

Mitochondria represent the major intracellular copper stock. Copper is essential for cytochrome C oxidase (COX) activity and mitochondrial respiration, but excess in mitochondrial copper may promote oxidative stress, impair electron transport chain function, and trigger cuproptosis under pathological conditions [27,28]. Genetic disorders of copper metabolism, such as Wilson’s and Menkes’ diseases, illustrate the neurotoxic consequences of severe copper imbalance [29].

The brain is particularly vulnerable to oxidative damage [30]. Physiologically, SODs, ceruloplasmin, GSH and MTs work as an efficient and integrated antioxidant defense system [28]. Copper homeostasis is also strictly linked to iron metabolism through ceruloplasmin, with its ferroxidase activity [31].

The convergence of synaptic copper signaling, mitochondrial redox activity, and protein quality control mechanisms suggests that even moderate copper imbalance may critically influence α-synuclein homeostasis.

## 3. Copper Dyshomeostasis and Clearance Pathway

The maintenance of protein homeostasis is essential for cellular functionality. The two primary pathways regulating protein catabolism are the UPS, deputed to the degradation of short-lived, soluble proteins, and the ALP, responsible for the degradation of long-lived proteins and organelles [11]. Impairment of both ALP and UPS can lead to the aggregation of unwanted proteins, and their cytotoxicity can lead to cell death. The two processes work dynamically; for instance, UPS inhibition has been shown to determine a compensatory activation of autophagy [11,32].

In neurodegenerative diseases, protein aggregates can cause damage by interfering with intracellular trafficking and sequestering proteins necessary for cell survival [33]. In contrast, some hypotheses suggest that large inclusion formations may have, initially, a cytoprotective role by sequestering toxic misfolded proteins [34].

### 3.1. Effects of Copper Dyshomeostasis on the Ubiquitin–Proteasome System

The UPS is the major pathway responsible for the selective degradation of cytosolic, nuclear, and endoplasmic reticulum proteins [35]. Proteins targeted for degradation are polyubiquitinated through an E1–E2–E3 enzymatic cascade and subsequently processed by the 26S proteasome in an ATP-dependent manner [36,37]. Several E3 ubiquitin ligases, including CHIP, E6AP and SIAH, regulate α-synuclein turnover, linking chaperone systems to proteasomal degradation [38,39].

In PD, the presence of ubiquitinated proteins in the LBs provides evidence of UPS involvement [40].

When the production of abnormal proteins overwhelms the proteasome, proteins accumulate in perinuclear structures resembling aggresomes, a phenomenon linked to α-synuclein inclusions [41].

These proteasome defects may derive from oxidative damage or reduced mitochondrial complex I activity in the SNpc. Since the protein ubiquitination process is ATP-dependent, mitochondrial impairment can directly lead to a UPS dysfunction [42]. Chronic administration of rotenone in rats confirmed this, resulting in the accumulation of α-synuclein/ubiquitin-positive inclusions in nigrostriatal dopaminergic neurons before neural degeneration [43,44].

Together, these observations indicate how copper homeostasis and UPS are closely associated. UPS controls copper uptake, efflux, and intracellular distribution by modulating CTR, ATP7A/B, COMMD1, and CCS, while copper availability reciprocally influences UPS activity by influencing ubiquitin-conjugating enzymes, deubiquitinases, and proteasomal function [45]. This bidirectional relationship provides a mechanistic framework for understanding how disturbances in both systems can synergistically amplify cellular stress and contribute to neurodegenerative processes.

In PD, copper dyshomeostasis has emerged as a significant pathogenic factor through its close interaction with UPS dysfunction and protein aggregation. While copper is essential for neuronal metabolism, inadequate buffering promotes oxidative stress, mitochondrial dysfunction, and protein misfolding, all of which are hallmarks of PD [46]. Both copper overload and deficiency negatively impact proteasomal activity: excess copper directly inhibits the 20S and 26S proteasomes as well as proteasome-associated deubiquitinases, whereas copper deficiency impairs the activity of copper-dependent antioxidant enzymes such as SOD1, increasing oxidative damage and indirectly saturating the UPS with misfolded substrates [47].

Impaired proteasomal activity leads to the accumulation and oligomerization of α-synuclein, which is normally degraded by the UPS [48]. Furthermore, copper binding to specific sites on the protein accelerates its misfolding and aggregation into toxic species that are inefficiently processed by the proteasome, thus amplifying proteotoxic stress [49].

Collectively, these findings indicate that in PD, copper imbalance and UPS dysfunction form a self-reinforcing pathogenic loop, promoting α-synuclein accumulation, oxidative stress and progressive dopaminergic neuron loss. Importantly, most inhibitory effects have been demonstrated under acute or high-dose copper exposure, raising questions about their relevance to the more subtle copper redistribution observed in PD brains. Thus, copper appears to function as a modulator of UPS efficiency rather than a primary initiator of proteasomal failure.

### 3.2. Effects of Copper Dyshomeostasis on the ALP

ALP is an evolutionary conserved process in which the intracellular components such as abnormal proteins, damaged organelles and other cellular components, are sequestered to be degraded [50,51]. In mammalian cells, the process is classified into three main types: macroautophagy, CMA and microautophagy. Macroautophagy provides the formation of a double-membrane organelle, the autophagosome [52], that works by delivering cargos to lysosomes. In contrast, in CMA and in microautophagy, the cargos are directly delivered to lysosomes [53]. CMA is a highly selective process in which substrates containing a KFERQ-like motif are recognized by Hsc70 and delivered to lysosomes via LAMP2A-mediated translocation [54]. Finally, microautophagy is a process in which cytoplasmic components, including peroxisomes, mitochondria, lipids, and the nucleus, enter the lysosome through invaginations or deformations of its membrane [55].

ALP efficiency is crucial for long-lived post-mitotic cells such as neurons, which are unable to dilute misfolded proteins or damaged organelles among daughter cells [56]. The first observation of an accumulation of autophagic vacuoles in the SNpc of PD patients dates to 1997 [57]. Experimental evidence suggests that impaired autophagic flux enhances α-synuclein toxicity [58].

Evidence from Wilson’s disease supports an association between copper imbalance and parkinsonism [59]. At high concentrations, copper induces toxicity through apoptosis and cuproptosis [28,60], despite also being able to trigger autophagy [61]. Excess copper promotes oxidative stress and mitochondrial dysfunction, two major inducers of autophagy in dopaminergic neurons. Chronic exposure to copper has been reported to promote the accumulation and aggregation of α-synuclein, a process accompanied by activation of autophagic pathways. In the A53T transgenic mouse model, even low copper concentrations further enhanced α-synuclein accumulation and Parkinsonian-like alterations, primarily through disruption of granular autophagy mechanisms [58].

Copper has been demonstrated to induce autophagy by increasing the expression of autophagy-related genes such as ATG5, SQSTM1/p62, and MAP1LC3 and by regulating key signaling pathways, including the AMPK-mTOR and PI3K/Akt/mTOR axes [62,63,64]. Mechanistically, copper can directly bind to and activate the autophagic kinases ULK1 and ULK2, promoting autophagy initiation independent of canonical upstream signaling [65,66]. In ATP7B-deficient cells, copper accumulation inhibits mTORC1 and activates the lysosomal biogenesis regulator TFEB, further promoting autophagic flux [67]. Interestingly, the effects of copper are dose dependent: while autophagy may initially protect cells, as observed in Wilson disease models [61], high copper concentrations, as well as chronic exposure to the metal can inhibit autophagy by interfering with essential ATG protease functions or promoting mTOR phosphorylation, compromising autophagic activities and favoring α-synuclein aggregation.

The main copper-dependent mechanisms involved in clearance are summarized in Table 1.

Overall, available evidence suggests that copper dyshomeostasis consistently affects the UPS than the ALP in PD. On the one hand, copper dyshomeostasis appears to directly impair the catalytic activity of the proteasome; on the other hand, copper’s impact on autophagy appears more complex and context-dependent. Although both systems contribute to α-synuclein turnover, current data more strongly support proteasome dysfunction as the primary target of copper imbalance, while autophagic alterations may represent secondary or compensatory responses.

## 4. Evidence of Copper Dyshomeostasis in PD

The imbalance of copper homeostasis has been identified as a critical factor in the development of idiopathic PD [81]. The increase of free copper is associated with increased oxidative stress, α-synuclein oligomerization and aggregation in LBs [82]. Copper ions can bind specifically to α-synuclein, contributing to its aggregation, potentially exacerbating pathology. In PD brains, decreased copper levels and altered ceruloplasmin activity have also been reported [82].

Experimental models show that systemic or intranigral copper exposure increases brain copper levels, reduces antioxidant defenses (e.g., GSH, SOD activity), and induces dopaminergic toxicity [83]. In humans, long-term exposure to copper in the workplace is also associated with an increased risk of developing PD [84]. The molecular link between copper dyshomeostasis and PD is not fully understood, but hypotheses have been suggested, which are based on some similarities of the clinical manifestations of PD with other copper-related diseases and on the ability of α-synuclein to bind specifically metal [8,85,86,87]. Another proposed mechanism is the ability of free copper to increase oxidative stress by catalyzing harmful redox reactions that include ROS [83] and decreases in GSH levels ranging from 40 to 90% in SNpc tissue in PD patients [88].

Both in vitro and in vivo studies have shown that copper can contribute to cellular toxicity [89,90]. The α-synuclein–copper complex exhibits redox activity, enhancing ROS production and promoting oxidation of dopamine and endogenous antioxidants such as GSH [9]. The copper–α-synuclein interaction can also induce relevant post-translational modifications in the same protein [91].

Although most studies associate high levels with an increased risk of developing PD, the hypothesis that a decrease in copper levels may be a risk factor is also supported. Several studies have highlighted that in patients with PD, the concentrations of copper and ceruloplasmin in the blood, in the locus coeruleus and in the substantia nigra, were lower than those in healthy subjects of the same age. By binding to ceruloplasmin, copper stimulates its ferroxidase activity and participates in iron homeostasis; therefore, it can be hypothesized that certain indirect toxicity mediated by altered iron concentrations may be a consequence of low copper levels [5].

However, the complexity of whole organisms, interindividual variability in clinical cohorts, and post-mortem confounding factors limit the ability to establish causality and to dissect specific clearance-related mechanisms. These limitations underscore the complementary role of in vitro models, which allow precise control over copper concentration, speciation, and intracellular compartmentalization. Evidence across experimental levels supports a hierarchical model in which in vivo and ex vivo studies establish pathological relevance, while in vitro models elucidate causative molecular mechanisms.

### 4.1. In Vitro Evidence

Immortalized cell lines represent the most widely used in vitro systems due to their ease of handling, genetic manipulability, and reproducibility [92].

Among these, human neuroblastoma-derived lines, particularly SH-SY5Y cells, are extensively employed for investigating PD pathology. SH-SY5Y cells can be differentiated into dopaminergic-like neurons and are highly amenable to genetic manipulation, including α-synuclein overexpression, making them suitable for mechanistic studies of synucleinopathies [93,94,95,96,97].

SH-SY5Y cells have been used to study metal-induced neurotoxicity, especially copper. Treatment with CuSO_4_ increases intracellular copper in a dose- and time-dependent manner, causing mitochondrial dysfunction, decreased membrane potential, impaired dehydrogenase activity, ROS production, and partial plasma membrane damage. These effects correlate with decreased mitochondrial proteins, including subunits of complexes I and V and the pyruvate dehydrogenase complex, highlighting mitochondria as early targets of copper-mediated oxidative stress that may trigger neurodegeneration [13,98].

Differentiated SH-SY5Y cells express higher levels of the copper transporter CTR1, enhancing copper uptake. Copper exposure increases α-synuclein phosphorylation via PLK2 upregulation, without affecting total α-synuclein or PP2A levels [99]. Additionally, copper induces synaptic loss, downregulates Wnt signaling, promotes β-catenin nuclear translocation, and disrupts autophagy (autophagosome accumulation, LC3B II/I and p62 increase) via inhibition of the PI3K/Akt/mTOR pathway, ultimately triggering apoptosis [12]. Pharmacological modulation shows that rapamycin alleviates, whereas chloroquine exacerbates, copper-induced neurotoxicity, highlighting autophagy’s critical role. Copper exposure also increases polyubiquitinated proteins, indicating UPS dysfunction, and promotes α-synuclein aggregation, especially phosphorylated forms localized in neurites, supporting copper’s role in α-synuclein pathology [12].

Neurotoxins such as 6-OHDA disrupt copper homeostasis by decreasing ATP7A and Atox1 levels, increasing intracellular copper and reducing dopamine β-hydroxylase, linking copper trafficking dysregulation to neuronal dysfunction [100].

Copper additionally modulates AMPK signaling. Low concentrations elevate p-AMPK, while high concentrations reduce it. AMPK inhibition worsens copper cytotoxicity, suggesting a protective role [101]. High copper also upregulates autophagy-related proteins and downregulates mTOR and p-ULK, inducing apoptosis [13].

Several neuroprotective compounds counteract copper toxicity in SH-SY5Y cells. Curcumin reduces ROS and MDA, restores mitochondrial potential, prevents cytochrome c translocation, modulates Bax/Bcl2 ratio, and decreases apoptosis [102]. Quercetin alleviates copper-induced oxidative and ER stress, mitochondrial dysfunction, and apoptosis while promoting autophagy via LC3-II upregulation, highlighting the therapeutic potential of antioxidants and autophagy modulators [103].

Rat pheochromocytoma PC12 cells are another widely used neuronal model to study metal-induced neurotoxicity and PD-related mechanisms [104]. NGF treatment differentiates PC12 cells into sympathetic neuron-like cells with dopaminergic features, including catecholamine synthesis, making them suitable for studying dopaminergic vulnerability [105,106]. A key pathogenic link involves 3,4-dihydroxyphenylacetaldehyde (DOPAL), a toxic dopamine metabolite that oligomerizes α-synuclein. In α-synuclein-overexpressing PC12 cells, divalent Cu(II) strongly enhances DOPAL-induced α-synuclein oligomerization, whereas Cu(I) and Fe(III) do not. This effect is attenuated by antioxidants and metal chelators, highlighting a synergy between dopamine metabolism and copper in α-synuclein aggregation [107].

Copper homeostasis critically influences α-synuclein fibrillation and cytotoxicity in PC12 cells. EGCG forms a Cu(II)/EGCG complex that inhibits α-synuclein conformational transitions, fibrillation, and aggregation, reduces ROS, and protects against copper-induced cytotoxicity, illustrating the therapeutic potential of chelation and antioxidants [108].

NGF-induced PC12 differentiation remodels copper handling: intracellular Cu increases, CTR1 expression decreases, brain-specific metallothionein MT-3 is induced, while MT-1/2 decrease, indicating adaptive neuronal Cu buffering. Differentiated neurons exhibit higher tolerance to Cu and cisplatin, and Cu(I)-dependent signaling via ERK1/2 is essential for neurite outgrowth and survival [109,110] Elevated Cu also alters vesicular trafficking, affecting neurotransmitter storage and exocytosis, linking copper dysregulation to PD-relevant processes [109,111,112].

LUHMES cells (Lund Human Mesencephalic cells), an immortalized human line, differentiate reliably into post-mitotic dopaminergic neurons expressing TH, DAT, and Nurr1, with robust neurite outgrowth, making them ideal for PD studies [110].

Exposure to CuSO_4_ causes cytotoxicity, lysosomal damage, early mitochondrial alterations, and network disruption at ≥100 µM, while glutathione depletion at ~300 µM impairs antioxidant defenses, linking copper dyshomeostasis to redox imbalance in human dopaminergic neurons [113].

Neuro-2a (N2A) cells, although less used in PD research, are employed to study copper-induced cytotoxicity and genotoxicity. Copper oxide nanoparticles induce mitochondrial dysfunction, lipid peroxidation, DNA fragmentation, and chromosomal damage via ROS-mediated pathways, demonstrating N2A sensitivity to copper-induced stress [114].

Non-neuronal human HEK293 and H4 cells are used to study copper’s cell-autonomous effects on α-synuclein aggregation and clearance. In HEK293 cells stably expressing fluorescent α-synuclein, CuSO_4_ treatment enhances inclusion formation in a dose-dependent manner, alters copper chaperones (CCS), and can be mitigated by CTR1 silencing [115,116].

While cellular systems provide mechanistic insight, their translational relevance is limited by the absence of glial buffering, systemic copper regulation, and physiological metal speciation. Therefore, in vitro findings should be interpreted as mechanistic hypotheses rather than direct reflections of in vivo pathophysiology.

### 4.2. Human Clinical and Animal Evidences

Post-mortem and biofluid studies consistently report altered copper distribution in PD, particularly within the substantia nigra [117,118,119]. Moreover, it has been found that these changes are frequent in probable preclinical disease cases, suggesting that changes in copper levels are probably related to early stages of the pathological process [120]. Indeed, this decrease is frequently accompanied by reduced ceruloplasmin expression or ferroxidase activity, impaired iron homeostasis and increased oxidative burden [121].

Altered iron and copper levels within the SN lead to increased oxidative stress, due to excess labile iron, as well as an inadequate cellular response to the increased oxidative load caused by dysfunctional and copper-deficient cuproproteins [122]. Combined, these two factors may represent key mechanisms contributing to neuronal death, but they also represent practical targets for next-generation therapies.

Ex vivo analyses further reveal abnormal copper distribution within PD brain tissue. Synchrotron-based X-ray fluorescence and histochemical studies have shown colocalization of copper with α-synuclein-positive LBs and neurites, supporting a direct interaction between copper and aggregated α-synuclein in PD brain [123,124]. In parallel, increased levels of redox-active copper have been detected in association with oxidative damage markers, including lipid peroxidation products and oxidatively modified proteins [125].

Clinical studies in cerebrospinal fluid and blood plasma have reported decreased ceruloplasmin-bound copper and a concomitant increase in non-ceruloplasmin-bound (“free”) copper in subsets of PD patients [81,126,127]. This shift toward labile copper species is particularly relevant, as free copper is capable of catalyzing Fenton-like reactions and directly binding α-synuclein, thereby promoting oxidative stress and protein misfolding. Importantly, elevated free copper levels have been correlated with disease severity and cognitive decline in some cohorts, although results remain heterogeneous due to differences in patient stratification, analytical techniques, and disease stage [81,128].

Multidimensional proteomic profiling revealed clusters of deregulated proteins related to copper and iron homeostasis in the SNpc of PD patients [117]. Meta-analyses of frozen brain parenchyma from patients with PD corroborate these findings, indicating a 45–65% reduction in copper levels in the SN, with comparable decreases in other affected regions, including the locus coeruleus [118]. These alterations occur in parallel with evidence of increased labile (non-protein-bound) copper species in biofluids, observed in specific subgroups of PD patients and associated with disease severity [119,121,129].

Collectively, human data supports the presence of copper redistribution rather than a uniform increase or decrease, with alterations that may depend on disease stage, brain region, and patient subgroup.

Animal studies have provided functional support for the pathological relevance of copper imbalance. Rodent models exposed to excess copper, either through dietary supplementation or direct intracerebral administration, exhibit increased oxidative stress, mitochondrial dysfunction, and selective degeneration of nigrostriatal dopaminergic neurons [130,131,132]. These models show enhanced α-synuclein aggregation, impaired motor performance, and reduced dopamine levels in the striatum [133,134].

Conversely, copper deficiency models also demonstrate increased neuronal vulnerability, emphasizing the narrow physiological window required for copper homeostasis [135]. Genetic manipulation of copper transport and handling pathways, including alterations in ATP7A, ATP7B, and ceruloplasmin, leads to dopaminergic dysfunction, increased oxidative stress, and impaired protein degradation systems in vivo [66,72]. Notably, several animal studies report reduced proteasomal activity and altered autophagic markers in response to copper perturbation, suggesting a direct impact of copper dyshomeostasis on protein clearance pathways [74].

Prolonged copper exposure in aging C57BL/6J mice induces dopaminergic neuron loss, motor deficits, α-synuclein accumulation, and alterations in protein clearance pathways, including increased ubiquitination and autophagy marker LC3BII/I, mirroring proteasomal and autophagic disruptions reported in vitro [124,136]. These findings support the hypothesis that copper dyshomeostasis drives both aggregation and clearance impairments in vivo.

In A53T α-synuclein transgenic mice, low-dose copper exposure exacerbates mitochondrial dysfunction, alters autophagy markers (e.g., LC3B II/I and p62), reduces the expression of dopaminergic markers (TH and DAT), increases oxidative stress, and promotes neuroinflammation, highlighting how copper imbalance interacts with genetic susceptibility to aggravate PD-related phenotype [123].

These findings support the concept that copper imbalance not only enhances α-synuclein aggregation but also interferes with its clearance pathways in vivo.

However, animal models also highlight important limitations. Systemic copper regulation, hepatic buffering, glial-mediated redistribution, and inflammatory responses can compensate for or obscure neuronal copper-specific alterations. Consequently, the direct molecular relationship between copper imbalance, α-synuclein accumulation, and autophagy or proteasome dysfunction is often indirect or inferred rather than directly demonstrated in vivo.

Although these findings link copper dyshomeostasis to PD, direct clinical evidence demonstrating a copper-dependent modulation of protein clearance pathways in patients is nowadays lacking. Despite the molecular interplay between copper, ALP and UPS is well characterized in in vitro systems, its relevance in human remains to be elucidated.

### 4.3. Strength, Gaps and Limitations

Despite substantial progress in understanding the role of copper overload in α-synuclein pathology, several critical gaps remain that limit the translation of in vitro findings to PD pathogenesis and therapy. A fundamental unresolved question is whether copper dyshomeostasis represents a primary pathogenic trigger or a secondary consequence of neurodegeneration. It is conceivable that α-synuclein aggregation itself disrupts intracellular copper distribution, establishing a self-amplifying vicious cycle between metal imbalance, oxidative stress, and proteostatic failure.

A major issue concerns the copper concentrations commonly employed in cell culture systems. Many studies use micromolar copper levels, which largely exceed the estimated physiological intracellular labile copper pools in neurons. While these concentrations are useful to elicit measurable toxic effects and dissect molecular pathways, they may not accurately reflect the chronic and subtle metal imbalance occurring in PD brains [137].

In vitro studies often employ copper concentrations that exceed physiological ranges, raising concerns about the relevance of observed effects. In most in vitro experiments, copper is administered as salt, CuSO_4_ or CuCl_2_, whereas in vivo copper is tightly bound to proteins and small ligands, such as ceruloplasmin, albumin, metallothioneins, and glutathione. The absence of physiological copper buffering systems in culture media may artificially enhance redox activity and oxidative stress, potentially overestimating copper-mediated toxicity and its impact on proteostasis [45,138].

Most in vitro studies focus on neuron-only systems, neglecting the contribution of non-neuronal cells to copper homeostasis and α-synuclein clearance. Astrocytes and microglia play key roles in copper uptake, storage, redistribution, and detoxification, as well as in the clearance of extracellular α-synuclein species [139]. Their absence represents a significant limitation, as it prevents the reproduction of the complex cellular interactions that regulate metal metabolism and proteostatic mechanisms in the brain [140]. Future directions should include co-culture systems, three-dimensional cultures, and brain organoids that better recapitulate cellular interactions and spatial organization relevant to copper metabolism and proteostasis.

Furthermore, many experimental models involve artificial overexpression of wild-type or mutant α-synuclein, which may not reflect physiological protein levels and can saturate degradation pathways independently of copper perturbations. This may confound the interpretation of results, making it difficult to discriminate between copper-specific effects and generic proteotoxic stress.

PD is a heterogeneous disorder with diverse genetic and environmental contributors. How copper dysregulation intersects with genetic risk factors, such as SNCA multiplication, LRRK2 mutations, or lysosomal gene variants, remains incompletely understood [141]. Emerging evidence suggests that gene–metal interactions may modulate vulnerability to proteostatic failure.

The identification of reliable biomarkers of copper dyshomeostasis and impaired α-synuclein clearance remains a major unmet need. Biomarkers reflecting labile copper pools, lysosomal dysfunction, or proteostatic stress could facilitate patient stratification and therapeutic monitoring [142,143]. Integrating mechanistic insights from in vitro models with biomarker-driven clinical studies may help bridge the gap between experimental findings and therapeutic development.

Overall, while in vitro systems remain indispensable for dissecting molecular mechanisms linking copper dyshomeostasis to impaired α-synuclein clearance, their translational relevance depends on careful experimental design, physiologically meaningful copper speciation and concentrations, and the integration of more complex models, such as co-cultures, three-dimensional systems, and patient-derived iPSC-based neurons [144].

In summary, current evidence supports a bidirectional model in which copper dyshomeostasis promotes α-synuclein aggregation and impairs its clearance, while proteostatic failure may further disrupt copper handling. Clarifying this interplay will be essential to determine whether modulation of copper homeostasis represents a viable therapeutic strategy in PD.

Together, these factors may limit the translational relevance of current cellular models and highlight the need for more complex and physiologically grounded experimental systems.

## 5. Therapeutic Applications

The growing body of evidence linking copper dyshomeostasis to impaired α-synuclein clearance highlights metal homeostasis as a potential therapeutic target in PD. Given the essential role of copper in neuronal metabolism, a critical distinction must be drawn between indiscriminate copper chelation and selective modulation of pathogenic copper pools [46,145].

### Copper Chelation and Metal-Targeting Approaches

Early therapeutic approaches relied on systemic copper chelation to mitigate oxidative stress and metal-induced toxicity. Although chelators have shown protective effects in selected experimental models, global copper depletion poses substantial risks, including impairment of mitochondrial respiration, antioxidant defense, and synaptic function [46,127,145]. Recent clinical and experimental evidence indicates that PD pathology is not driven by increased total copper levels, but rather by altered intracellular distribution and expansion of labile, redox-active copper pools [46,146].

Accordingly, therapeutic strategies aimed at buffering or redistributing intracellular copper, rather than removing it, are increasingly favored.

Several clinical studies reports decreased circulating copper levels in PD patients compared to healthy controls [147]. Because copper binding to ceruloplasmin stimulates ferroxidase activity and contributes to iron homeostasis, reduced copper availability may indirectly promote iron accumulation and oxidative toxicity [148]. These findings underscore the complexity of copper biology in PD and caution against indiscriminate complete copper chelation strategies, being copper metabolism coupled to those of iron.

In preclinical PD models, chelation of heavy metals including copper and iron improved motor and non-motor deficits following MPTP treatment [149]. However, these experimental benefits have not translated into convincing disease-modifying effects in PD patients.

Several copper-chelating compounds have been shown in vitro to attenuate the effects by reducing copper-induced α-synuclein aggregation, oxidative damage, and cell death in dopaminergic neuronal systems [26,150]. Classical copper chelators such as D-penicillamine and trientine efficiently sequester copper but are poorly suited for PD due to limited BBB permeability and the risk of indiscriminate disruption of essential copper-dependent processes [151].

In addition, these compounds may pose several adverse effects; it is the case of trientine, whose overdose comes with nausea, dizziness and sideroblastic anemia [152]. However, its relevance to PD remains limited, as it exhibits poor BBB penetration. Recent strategies are exploring targeted delivery systems, such as D-penicillamine-loaded MIL-100(Fe) nanocarriers, to improve the specificity and efficacy of copper chelation [153] and surface-modified liposomes, to enhance the transport of TETA across the blood–brain barrier and improve targeted copper chelation [154].

Consequently, attention has shifted toward moderate-affinity chelators and metal-protein attenuating compounds (MPACs), including 8-hydroxyquinoline derivatives and multifunctional ligands, which in vitro redistribute copper away from pathological protein-binding sites without inducing complete metal depletion [155,156,157]. MPACs are able to overcome the BBB and have emerged as promising therapeutic strategies.

These compounds have been reported to reduce copper-mediated α-synuclein toxicity, preserve mitochondrial function, and improve neuronal viability [99,158]. It remains unclear whether copper-targeting interventions would be most effective at early, pre-symptomatic stages, when metal redistribution and proteostatic stress begin, or in established disease, where neuronal loss is already extensive.

Collectively, these findings support a conservative and context-dependent model of copper chelation in PD, reinforcing its potential role as part of multitarget neuroprotective strategies rather than as a standalone disease-modifying intervention [159,160].

Clinical translation of metal-targeting strategies in PD remains limited, and available trials have yielded mixed results, underscoring the need for biomarker-guided patient stratification.

Overall, therapeutic modulation of copper in PD should aim to restore physiological metal compartmentalization rather than achieve systemic decoppering. A precision-based approach integrating biomarkers of labile copper pools and proteostatic dysfunction may help identify patient subgroups most likely to benefit from metal-targeting strategies.

Despite growing preclinical interest in copper-modulating strategies, controlled clinical trials specifically targeting copper chelation in PD are currently lacking.

Compounds such as PBT2 and other 8-hydroxyquinoline derivatives have been evaluated in neurodegenerative settings, primarily in Alzheimer’s disease, where they demonstrated acceptable tolerability but limited and inconclusive efficacy [161,162].

Most clinical experience with metal chelation in PD comes from studies targeting iron accumulation, such as deferiprone, rather than from interventions specifically designed to modulate copper homeostasis [163,164]. Therefore, while experimental models suggest that redistributing labile copper pools can attenuate protein aggregation and proteostatic dysfunction, the translational potential of copper-targeted chelation strategies in PD remains largely untested. Future clinical investigations will require careful patient stratification, biomarker-guided selection, and precise modulation of intracellular copper pools to avoid systemic copper depletion and preserve metal-dependent physiological functions.

An overview of copper-chelating strategies and their mechanistic implications is reported in Table 2.

## 6. Future Challenges and Perspectives

A major challenge in this field is reconciling the robust mechanistic evidence obtained from in vitro models with the heterogeneous and sometimes conflicting observations reported in in vivo and ex vivo studies [5,8]. While animal models and post-mortem human tissues have been essential to establish the relevance of copper dyshomeostasis, α-synuclein pathology, and autophagy deficiency in PD, the intrinsic complexity of the whole organism often hides direct cause–effect relationships [176]. Systemic metal redistribution, compensatory responses, neuroinflammation, and circuit-level adaptations can hide the key molecular events linking copper imbalance to the failure of α-synuclein clearance. In this context, in vitro systems have proven indispensable for isolating the direct copper–α-synuclein–proteostasis axis and demonstrating that copper acts as an active modulator of clearance pathways rather than a nonspecific toxicant [177].

Data derived from human clinical studies, animal models, and ex vivo analyses consistently indicate that copper homeostasis is altered in PD, although these alterations rarely manifest as uniform changes in total copper levels. Instead, converging findings point toward a pathological redistribution of copper into poorly buffered, redox-active pools that critically affect neuronal vulnerability [178].

Nevertheless, conventional cellular models also present important limitations, including reduced cellular diversity, altered metabolic states, and incomplete representation of neuronal–glial interactions that are critical for copper buffering and protein homeostasis in the brain. Future research should therefore move toward more advanced human-relevant systems that retain experimental controllability while increasing biological complexity [179]. Induced pluripotent stem cells-derived dopaminergic (iPSC-DA) neurons offer a powerful platform to investigate copper dyshomeostasis in a genetically defined human context, enabling the study of gene–metal interactions relevant to PD [180,181,182]. Three-dimensional (3D) culture systems, including spheroids and iPSC-based brain organoids, represent a further level of complexity by recapitulating aspects of tissue architecture, cell–cell communication, and spatial metal distribution [183,184,185,186]. These models recapitulate aspects of tissue architecture, cell–cell communication, and spatial metal gradients that are inaccessible in 2D cultures [183,187,188]. Recent work demonstrates that organoids can model progressive α-synuclein pathology, lysosomal dysfunction, and mitochondrial stress over extended time frames, making them particularly suited for studying chronic processes such as metal dyshomeostasis [185,188,189]. Importantly, these systems allow investigation of copper distribution and compartmentalization at the tissue-like level, a key unresolved issue in PD research.

The integration of co-culture systems incorporating astrocytes and microglia will be particularly important to capture non-cell-autonomous mechanisms of copper handling and α-synuclein clearance [178,190].

Ultimately, the challenge will be to integrate data across multiple levels of biological complexity. Rather than replacing in vivo studies, advanced in vitro models should be viewed as a critical intermediate step that preserves mechanistic resolution while improving translational relevance.

**Figure 1:**
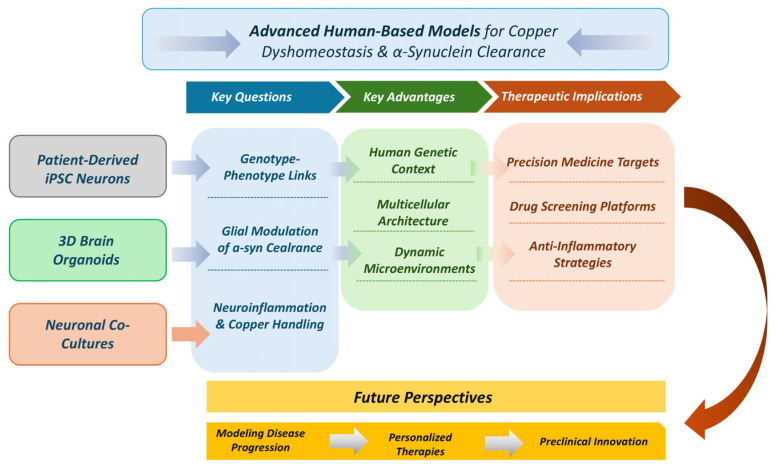
Schematic representation of advanced human-based models for the study of copper dyshomeostasis and α-synuclein clearance in PD. iPSC-derived neurons, 3D-brain organoids and co-cultures systems could provide advantages over conventional systems by preserving genetic context, multicellular architecture, and dynamic microenvironments. Arrows indicate the progression from model systems to mechanistic insights and applications.

From an ethical and regulatory perspective, the refinement and implementation of advanced in vitro models align with the principles of reduction and refinement of animal experimentation. Addressing mechanistic questions in human-relevant cellular systems may reduce the need for animal studies in cases where equivalent or superior mechanistic insight can be achieved, while preserving in vivo models for questions that truly require organismal complexity, such as behavioral outcomes or systemic pharmacokinetics [191,192]. The combined use of 2D cellular systems, iPSC-based co-cultures, and 3D organoids, together with targeted in vivo validation, represents a rational and scalable framework to elucidate the role of copper dyshomeostasis in α-synuclein clearance and to identify therapeutic strategies aimed at restoring proteostasis in PD. A schematic representation of advanced human-based models for studying copper dyshomeostasis is shown in Figure 1.

## 7. Conclusions

In conclusion, copper dyshomeostasis emerges as a multifaceted contributor to PD pathogenesis, acting not only through direct effects on α-synuclein aggregation, but also by disrupting the cellular machinery responsible for protein clearance. Most mechanistic insights come from in vitro models, which have been instrumental in analyzing the early molecular events linking copper exposure to α-synuclein aggregation and proteostatic stress. Evidence from animal models and human studies partially supports the presence of altered copper distribution and redox imbalance in vulnerable brain regions in PD.

While significant challenges remain, particularly in translation and patient stratification, advances in human-based cellular models and targeted therapeutic strategies offer a promising path forward. From a therapeutic perspective, strategies aimed at modulating intracellular copper pools, rather than inducing systemic copper depletion, represent a promising conceptual approach. However, clinical evidence supporting copper-targeted interventions in PD is still lacking, and the development of effective therapies will require a better understanding of the temporal dynamics and cellular specificity of copper dysregulation.

## Figures and Tables

**Table 1:** Effects of copper on α-synuclein clearance mechanisms.

Clearance Pathway	Copper-Related Effect	Mechanism	Outcome on α-Synuclein	References
UPS	Proteasome inhibition	Oxidative modification of proteasomal subunits, reduced catalytic activity	Accumulation of soluble α-synuclein, impaired degradation	[14,49,68,69]
Macroautophagy	Autophagic flux blockade	Impaired autophagosome formation/maturation, lysosomal dysfunction	Persistence of α-synuclein aggregates, impaired clearance	[12,13,70,71]
CMA	LAMP2A interference	Copper-induced α-synuclein modifications impair LAMP2A recognition	Failure of selective α-synuclein clearance	[72,73,74]
Mitophagy	Indirect inhibition	Mitochondrial stress, ATP depletion	Proteostasis imbalance, secondary α-synuclein accumulation	[75,76,77,78,79,80]

**Table 2:** Copper-chelating strategies in PD.

Chelating Agent/Class	Metal Target(s)	Experimental Model (In Vitro/In Vivo/Preclinical)	Main Effects Relevant to PD	Mechanistic Implications	References
D-Penicillamine	Cu(I), Cu(II)	In vitro; in vivo	Efficient copper sequestration and reduction of copper availability; modulation of copper-induced oxidative damage and apoptosis	Non-selective copper removal; modulation of apoptotic pathways (Bax/Bcl-2 balance) under copper-induced toxicity; limited CNS applicability due to poor BBB penetration	[151,153,165]
Trientine (TETA)	Cu(I), Cu(II), Fe(II), Zn(II)	In vitro; in vivo	Reduction of bioavailable copper; antioxidant effects	Systemic decoppering; potential interference with physiological copper-dependent enzymes; hydrophilic nature limiting BBB penetration	[154,166,167]
Tetrathiomolybdate (TM)	Cu(I), Cu(II)	In vitro; in vivo	Strong copper chelation; reduction of copper-mediated oxidative stress	Excessive copper depletion; limited applicability in PD	[168,169,170]
8-Hydroxyquinoline derivatives	Cu(II), Fe(II/III), Zn(II)	In vitro;	Reduction of Cu-induced α-synuclein aggregation; improved cell viability	Metal redistribution rather than bulk chelation; moderate affinity	[155,156,157]
PBT2 (MPAC)	Cu(II), Zn(II)	In vitro; in vivo	Attenuation of metal–α-synuclein interactions; reduced oxidative stress	Metal–protein attenuation; preservation of physiological metal pools	[171,172]
Deferiprone	Fe(III), Fe(II); Cu(II)	In vivo	Reduction of redox-active metal pools; neuroprotection	Dual iron/copper modulation; indirect effects on copper homeostasis	[173,174,175]

## Data Availability

No new data were created or analyzed in this study. Data sharing is not applicable to this article.

## Abbreviations

AD	Alzheimer’s Disease
AKT	serine/threonine protein kinase
ALP	Autophagy–Lysosome Pathway
AMPK	AMP-activated Protein Kinase
APP	Amyloid Precursor Protein
ATGs	Autophagy-related family proteins
ATP7A	ATPase copper Transporting Alpha
ATP7B	ATPase copper Transporting Beta
BBB	Blood–Brain Barrier
BCB	Blood–Cerebrospinal fluid Barrier
BDNF	Brain-Derived Neurotrophic Factor
CCS	Copper Chaperone for SOD1
CMA	Chaperone-Mediated Autophagy
COX	Cytochrome C Oxidase
CTR1	Copper Transporter 1
DA	Dopamine
DMT1	Divalent Metal Transporter 1
ETC	Electron Transport Chain
GBA-1	Glucosylceramidase Beta 1
GPx	Glutathione Peroxidase
GRKs	G protein-coupled Receptor Kinases
GSH	Glutathione
HSPs	Heat Shock Proteins
IMM	Inner Mitochondrial Membrane
IMS	Intermembrane Space
LAMP2A	Lysosomal-Associated Membrane Protein 2A
LBs	Lewy Bodies
L-DOPA	Levodopa
LRRK2	Leucine-rich repeat kinase 2
MAPT	Microtubule Associated Protein Tau
MPP+	1-methyl-4-phenylpyridinium +
MPTP	1-methyl-4-phenyl-1,2,3,6-tetrahydropyridine
mTOR	mammalian Target of Rapamycin
MTs	Metallothioneins
NAC	Non-Amyloid beta Component
NADPH	Nicotinamide Adenine Dinucleotide Phosphate
NGF	Nerve Growth Factor
NMDA	N-Methyl-D-Aspartate
OMM	Outer Mitochondrial Membrane
PAM	Peptidylglycine α-Amidating Monooxygenase
PD	Parkinson’s Disease
PI3K	Phosphatidyl-Inositol 3-Kinase
PINK1	PTEN Induced Kinase 1
PLKs	Polo-Like Kinases
PrP	Prion Protein
PTMs	Post-Translational Modifications
RNS	Reactive Nitrogen Species
ROS	Reactive Oxygen species
SCO1	cytochrome c oxidase assembly protein 1
SCO2	cytochrome c oxidase assembly protein 2
SNARE	Soluble N-ethylmaleimide Attachment protein Receptor
